# (*E*)-1-(2,4-Dinitro­phen­yl)-2-[1-(2-meth­oxy­phen­yl)ethyl­idene]hydrazine

**DOI:** 10.1107/S1600536811045417

**Published:** 2011-11-05

**Authors:** Hoong-Kun Fun, Boonlerd Nilwanna, Patcharaporn Jansrisewangwong, Thawanrat Kobkeatthawin, Suchada Chantrapromma

**Affiliations:** a X-ray Crystallography Unit, School of Physics, Universiti Sains Malaysia, 11800 USM, Penang, Malaysia; b Crystal Materials Research Unit, Department of Chemistry, Faculty of Science, Prince of Songkla University, Hat-Yai, Songkhla 90112, Thailand

## Abstract

The mol­ecule of the title compound, C_15_H_14_N_4_O_5_, is in an *E* conformation with respect to the C=N double bond and the dihedral angle between the two benzene rings is 37.83 (7)°. The ethyl­idenehydrazine plane makes dihedral angles of 4.93 (9) and 42.38 (9)° with the two benzene rings. An intra­molecular N—H⋯O hydrogen bond generates an *S*(6) ring motif. In the crystal, mol­ecules are linked by weak C—H⋯O inter­actions into chains along the *c* axis which are stacked along the *b* axis by aromatic π–π inter­actions with a centroid–centroid distance of 3.5927 (10) Å.

## Related literature

For bond-length data, see: Allen *et al.* (1987 ▶). For hydrogen-bond motifs, see: Bernstein *et al.* (1995 ▶). For related structures see: Fun *et al.* (2011 ▶); Jansrisewangwong *et al.* (2010 ▶); Nilwanna *et al.* (2011 ▶). For background to the biological activity of hydro­zones, see: Bendre *et al.* (1998 ▶); Cui *et al.* (2010 ▶); Gokce *et al.* (2009 ▶); Khan *et al.* (2007 ▶); Loncle *et al.* (2004 ▶); Wang *et al.* (2009 ▶).
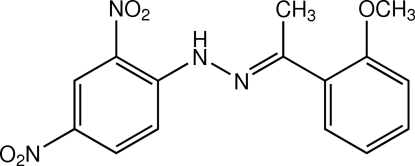


## Experimental

### 

#### Crystal data

C_15_H_14_N_4_O_5_
*M* *_r_* = 330.30
Monoclinic, 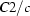

*a* = 33.105 (5) Å
*b* = 7.1288 (10) Å
*c* = 13.4964 (19) Å
β = 107.170 (2)°
*V* = 3043.2 (8) Å^3^
*Z* = 8
Mo *K*α radiation
μ = 0.11 mm^−1^
*T* = 297 K
0.35 × 0.33 × 0.21 mm

#### Data collection

Bruker APEXII CCD diffractometer
Absorption correction: multi-scan (*SADABS*; Bruker, 2005 ▶) *T*_min_ = 0.962, *T*_max_ = 0.977
11675 measured reflections
4013 independent reflections
2945 reflections with *I* > 2σ(*I*)
*R*_int_ = 0.019

#### Refinement

*R*[*F* ^2^ > 2σ(*F* ^2^)] = 0.042
*wR*(*F* ^2^) = 0.124
*S* = 1.04
4013 reflections
223 parameters
H atoms treated by a mixture of independent and constrained refinement
Δρ_max_ = 0.19 e Å^−3^
Δρ_min_ = −0.17 e Å^−3^

### 

Data collection: *APEX2* (Bruker, 2005 ▶); cell refinement: *SAINT* (Bruker, 2005 ▶); data reduction: *SAINT*; program(s) used to solve structure: *SHELXTL* (Sheldrick, 2008 ▶); program(s) used to refine structure: *SHELXTL*; molecular graphics: *SHELXTL*; software used to prepare material for publication: *SHELXTL* and *PLATON* (Spek, 2009 ▶).

## Supplementary Material

Crystal structure: contains datablock(s) global, I. DOI: 10.1107/S1600536811045417/hb6478sup1.cif

Structure factors: contains datablock(s) I. DOI: 10.1107/S1600536811045417/hb6478Isup2.hkl

Supplementary material file. DOI: 10.1107/S1600536811045417/hb6478Isup3.cml

Additional supplementary materials:  crystallographic information; 3D view; checkCIF report

## Figures and Tables

**Table 1:** Hydrogen-bond geometry (Å, °)

*D*—H⋯*A*	*D*—H	H⋯*A*	*D*⋯*A*	*D*—H⋯*A*
N1—H1*N*1⋯O1	0.87 (2)	1.952 (18)	2.6086 (17)	131.1 (15)
C6—H6*A*⋯O3^i^	0.93	2.48	3.218 (2)	136

Symmetry code: (i) 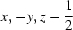.

**Fig. 1.**
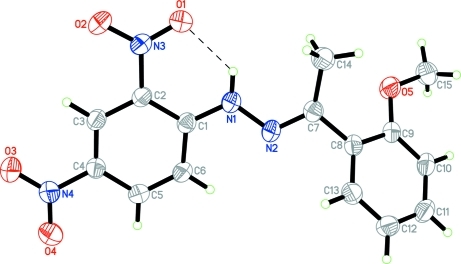
The molecular structure of (I), showing 40% probability displacement ellipsoids. Hydrogen bond is shown as a dashed line.

**Fig. 2.**
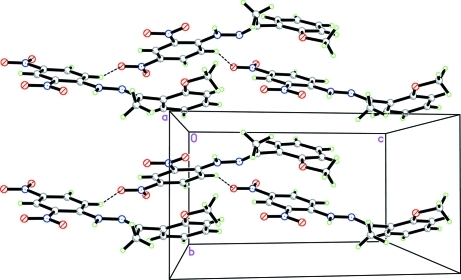
The crystal packing of (I) viewed approximately along the a axis, showing chains stacked along the b axis. Hydrogen bonds are shown as dashed lines.

## supplementary crystallographic information

### Comment

For a long time, hydrazone derivatives have been studied for their biological properties such as antibacterial, antioxidant, antitumor, antifungal, analgesic and anti-inflammatory (Cui *et al.*, 2010; Gokce *et al.*, 2009; Khan *et al.*, 2007; Loncle *et al.*, 2004; Wang *et al.*, 2009) and tyrosinase inhibitory activities (Bendre *et al.*, 1998). In our previous studies, we reported the syntheses and crystal structures of some hydrazone derivatives (Fun *et al.*, 2011; Jansrisewangwong *et al.*, 2010); Nilwanna *et al.*, 2011). The title compound (I) was designed and synthesized in order to study its bioactivity properties. It has been screened for antibacterial activity but found to be inactive.

The molecule of (I) (Fig. 1), C_15_H_14_N_4_O_5_, is twisted and exists in an *E* configuration with respect to the ethylidene C═N double bond [1.2845 (17) Å] with the torsion angle N1–N2–C7–C8 = 176.97 (11)°. The dihedral angle between the two benzene rings is 37.83 (7)°. The ethylidenehydrazine fragment is planar with the *r.m.s* deviation of 0.0027 (1) Å and the torsion angle N1–N2–C7–C14 = 0.9 (2)°. This middle C/C/N/N plane makes the dihedral angles of 4.93 (9) and 42.38 (9)° with the 2,4-dinitrophenyl and 2-methoxyphenyl rings, respectively. The two nitro groups of 2,4-dinitrophenyl are co-planar with the bound benzene ring with the *r.m.s*. deviation of 0.0124 (1) Å for the twelve non H-atoms. In addition the methoxy group is almost co-planar with its attached benzene ring with the torsion angle C15–O5–C9–C10 = -6.2 (2)°. Intramolecular N1—H1···O1 hydrogen bond (Fig. 1 and Table 1) generates an S(6) ring motif (Bernstein *et al.*, 1995). The bond distances are within the normal range (Allen *et al.*, 1987) and are comparable with the related structures (Fun *et al.*, 2011; Jansrisewangwong *et al.*, 2010; Nilwanna *et al.*, 2011).

In the crystal structure (Fig. 2), the molecules are linked by C—H···O weak interactions (Table 1) into chains along the *c* axis. These chains are stacked along the *b* axis by π–π interaction with the *Cg*_1_···*Cg*_2_ distance = 3.5927 (10) Å (symmetry code: x, -y, 1/2+z); *Cg*_1_ and *Cg*_2_ are the centroids of C1–C6 and C8–C13 benzene rings, respectively.

### Experimental

The title compound (I) was synthesized by dissolving  2,4-dinitrophenylhydrazine (0.40 g, 2 mmol) in ethanol (10.00 ml) and H_2_SO_4_ (conc.) (98 %, 0.50 ml) was slowly added with stirring. 2-methoxyacetophenone (0.30 ml, 2 mmol) was then added to the solution with continuous stirring. The solution was refluxed for 1 hr yielding an orange solid, which was filtered off and washed with methanol. Orange blocks were recrystalized from ethanol by slow evaporation of the solvent at room temperature over several days, Mp. 462-463 K.

### Refinement

Amide H atom was located in a Fourier difference map and refined isotropically. The remainning H atoms were positioned geometrically and allowed to ride on their parent atoms, with d(C-H) = 0.93 Å for aromatic and 0.96 Å for CH_3_ atoms. The *U*_iso_ values were constrained to be 1.5*U*_eq_ of the carrier atom for methyl H atoms and 1.2*U*_eq_ for the remaining H atoms. A rotating group model was used for the methyl groups.

### Crystal data

**Table:**

C_15_H_14_N_4_O_5_	*F*(000) = 1376
*M**_r_* = 330.30	*D*_x_ = 1.442 Mg m^−^^3^
Monoclinic, *C*2/*c*	Melting point = 462–463 K
Hall symbol: -C 2yc	Mo *K*α radiation, λ = 0.71073 Å
*a* = 33.105 (5) Å	Cell parameters from 4013 reflections
*b* = 7.1288 (10) Å	θ = 2.6–29.0°
*c* = 13.4964 (19) Å	µ = 0.11 mm^−^^1^
β = 107.170 (2)°	*T* = 297 K
*V* = 3043.2 (8) Å^3^	Block, orange
*Z* = 8	0.35 × 0.33 × 0.21 mm

### Data collection

**Table:**

Bruker APEXII CCD diffractometer	4013 independent reflections
Radiation source: sealed tube	2945 reflections with *I* > 2σ(*I*)
graphite	*R*_int_ = 0.019
φ and ω scans	θ_max_ = 29.0°, θ_min_ = 2.6°
Absorption correction: multi-scan (*SADABS*; Bruker, 2005)	*h* = −44→43
*T*_min_ = 0.962, *T*_max_ = 0.977	*k* = −7→9
11675 measured reflections	*l* = −18→18

### Refinement

**Table:**

Refinement on *F*^2^	Primary atom site location: structure-invariant direct methods
Least-squares matrix: full	Secondary atom site location: difference Fourier map
*R*[*F*^2^ > 2σ(*F*^2^)] = 0.042	Hydrogen site location: inferred from neighbouring sites
*wR*(*F*^2^) = 0.124	H atoms treated by a mixture of independent and constrained refinement
*S* = 1.04	*w* = 1/[σ^2^(*F*_o_^2^) + (0.0564*P*)^2^ + 1.0718*P*] where *P* = (*F*_o_^2^ + 2*F*_c_^2^)/3
4013 reflections	(Δ/σ)_max_ = 0.001
223 parameters	Δρ_max_ = 0.19 e Å^−^^3^
0 restraints	Δρ_min_ = −0.17 e Å^−^^3^

### Special details

**Table:**

Geometry. All esds (except the esd in the dihedral angle between two l.s. planes) are estimated using the full covariance matrix. The cell esds are taken into account individually in the estimation of esds in distances, angles and torsion angles; correlations between esds in cell parameters are only used when they are defined by crystal symmetry. An approximate (isotropic) treatment of cell esds is used for estimating esds involving l.s. planes.
Refinement. Refinement of F^2^ against ALL reflections. The weighted R-factor wR and goodness of fit S are based on F^2^, conventional R-factors R are based on F, with F set to zero for negative F^2^. The threshold expression of F^2^ > 2sigma(F^2^) is used only for calculating R-factors(gt) etc. and is not relevant to the choice of reflections for refinement. R-factors based on F^2^ are statistically about twice as large as those based on F, and R- factors based on ALL data will be even larger.

### Fractional atomic coordinates and isotropic or equivalent isotropic displacement parameters (Å^2^)

**Table:**

	*x*	*y*	*z*	*U*_iso_*/*U*_eq_	
O1	0.07338 (3)	0.25310 (17)	0.98816 (8)	0.0590 (3)	
O2	0.10350 (4)	0.20839 (19)	1.15077 (8)	0.0675 (3)	
O3	0.24539 (4)	−0.0059 (3)	1.28175 (9)	0.0877 (5)	
O4	0.28280 (4)	−0.0482 (2)	1.17821 (10)	0.0851 (4)	
O5	0.03857 (3)	0.18362 (17)	0.48812 (7)	0.0560 (3)	
N1	0.11540 (4)	0.21520 (17)	0.85348 (8)	0.0444 (3)	
H1N1	0.0924 (6)	0.258 (2)	0.8644 (13)	0.057 (5)*	
N2	0.12306 (4)	0.22293 (16)	0.75869 (8)	0.0433 (3)	
N3	0.10449 (4)	0.20867 (17)	1.06074 (8)	0.0455 (3)	
N4	0.24971 (4)	−0.0049 (2)	1.19497 (10)	0.0585 (3)	
C1	0.14719 (4)	0.16224 (18)	0.93741 (9)	0.0385 (3)	
C2	0.14337 (4)	0.15613 (19)	1.03938 (9)	0.0390 (3)	
C3	0.17682 (4)	0.10245 (19)	1.12370 (9)	0.0429 (3)	
H3A	0.1738	0.1001	1.1900	0.052*	
C4	0.21432 (4)	0.0530 (2)	1.10768 (10)	0.0446 (3)	
C5	0.21945 (4)	0.0566 (2)	1.00856 (10)	0.0489 (3)	
H5A	0.2451	0.0222	0.9991	0.059*	
C6	0.18673 (4)	0.1105 (2)	0.92606 (10)	0.0469 (3)	
H6A	0.1905	0.1134	0.8605	0.056*	
C7	0.09330 (4)	0.28669 (19)	0.68182 (9)	0.0412 (3)	
C8	0.10499 (4)	0.29961 (18)	0.58364 (9)	0.0399 (3)	
C9	0.07723 (4)	0.24861 (19)	0.48709 (9)	0.0415 (3)	
C10	0.09020 (5)	0.2587 (2)	0.39813 (10)	0.0482 (3)	
H10A	0.0718	0.2241	0.3343	0.058*	
C11	0.13047 (5)	0.3199 (2)	0.40454 (11)	0.0545 (4)	
H11A	0.1390	0.3269	0.3449	0.065*	
C12	0.15792 (5)	0.3706 (2)	0.49859 (12)	0.0546 (4)	
H12A	0.1850	0.4119	0.5026	0.066*	
C13	0.14525 (4)	0.3602 (2)	0.58725 (11)	0.0474 (3)	
H13A	0.1641	0.3945	0.6506	0.057*	
C14	0.05141 (5)	0.3552 (3)	0.68823 (11)	0.0571 (4)	
H14A	0.0558	0.4461	0.7429	0.086*	
H14B	0.0355	0.2515	0.7024	0.086*	
H14C	0.0361	0.4121	0.6235	0.086*	
C15	0.00827 (5)	0.1403 (3)	0.39214 (12)	0.0672 (5)	
H15A	−0.0176	0.1009	0.4043	0.101*	
H15B	0.0189	0.0410	0.3586	0.101*	
H15C	0.0031	0.2495	0.3486	0.101*	

### Atomic displacement parameters (Å^2^)

**Table:**

	*U*^11^	*U*^22^	*U*^33^	*U*^12^	*U*^13^	*U*^23^
O1	0.0455 (5)	0.0817 (8)	0.0507 (6)	0.0075 (5)	0.0154 (5)	0.0039 (5)
O2	0.0637 (7)	0.1012 (9)	0.0455 (6)	0.0124 (6)	0.0285 (5)	0.0024 (6)
O3	0.0624 (7)	0.1543 (14)	0.0456 (6)	0.0142 (8)	0.0147 (5)	0.0277 (7)
O4	0.0485 (6)	0.1397 (13)	0.0658 (8)	0.0201 (7)	0.0149 (6)	0.0156 (8)
O5	0.0498 (6)	0.0784 (8)	0.0372 (5)	−0.0142 (5)	0.0087 (4)	−0.0049 (5)
N1	0.0461 (6)	0.0533 (7)	0.0342 (5)	0.0014 (5)	0.0127 (5)	−0.0016 (5)
N2	0.0496 (6)	0.0477 (6)	0.0325 (5)	−0.0008 (5)	0.0121 (4)	−0.0010 (4)
N3	0.0464 (6)	0.0521 (7)	0.0414 (6)	−0.0002 (5)	0.0183 (5)	−0.0005 (5)
N4	0.0457 (7)	0.0816 (10)	0.0463 (6)	0.0003 (6)	0.0106 (5)	0.0125 (6)
C1	0.0426 (6)	0.0387 (7)	0.0342 (6)	−0.0055 (5)	0.0112 (5)	−0.0019 (5)
C2	0.0415 (6)	0.0413 (7)	0.0365 (6)	−0.0044 (5)	0.0153 (5)	−0.0021 (5)
C3	0.0465 (7)	0.0486 (8)	0.0352 (6)	−0.0058 (6)	0.0143 (5)	0.0012 (5)
C4	0.0415 (7)	0.0525 (8)	0.0386 (6)	−0.0056 (6)	0.0098 (5)	0.0038 (5)
C5	0.0421 (7)	0.0627 (9)	0.0449 (7)	−0.0013 (6)	0.0172 (6)	0.0012 (6)
C6	0.0470 (7)	0.0595 (9)	0.0374 (6)	−0.0027 (6)	0.0173 (5)	−0.0012 (6)
C7	0.0452 (7)	0.0411 (7)	0.0361 (6)	−0.0033 (5)	0.0101 (5)	−0.0048 (5)
C8	0.0442 (7)	0.0388 (7)	0.0357 (6)	0.0040 (5)	0.0104 (5)	0.0025 (5)
C9	0.0458 (7)	0.0414 (7)	0.0356 (6)	0.0027 (6)	0.0094 (5)	0.0033 (5)
C10	0.0583 (8)	0.0498 (8)	0.0353 (6)	0.0055 (6)	0.0120 (6)	0.0043 (5)
C11	0.0655 (9)	0.0573 (9)	0.0475 (7)	0.0078 (7)	0.0272 (7)	0.0096 (6)
C12	0.0481 (8)	0.0565 (9)	0.0630 (9)	0.0004 (7)	0.0225 (7)	0.0094 (7)
C13	0.0448 (7)	0.0478 (8)	0.0465 (7)	−0.0003 (6)	0.0088 (6)	0.0031 (6)
C14	0.0512 (8)	0.0737 (11)	0.0454 (7)	0.0067 (7)	0.0128 (6)	−0.0095 (7)
C15	0.0522 (9)	0.0965 (14)	0.0443 (8)	−0.0065 (9)	0.0012 (7)	−0.0053 (8)

### Geometric parameters (Å, °)

**Table:**

O1—N3	1.2347 (15)	C6—H6A	0.9300
O2—N3	1.2251 (14)	C7—C8	1.4889 (16)
O3—N4	1.2212 (16)	C7—C14	1.497 (2)
O4—N4	1.2215 (17)	C8—C13	1.3880 (19)
O5—C9	1.3649 (17)	C8—C9	1.4029 (17)
O5—C15	1.4186 (17)	C9—C10	1.3914 (17)
N1—C1	1.3526 (17)	C10—C11	1.381 (2)
N1—N2	1.3768 (14)	C10—H10A	0.9300
N1—H1N1	0.871 (18)	C11—C12	1.373 (2)
N2—C7	1.2845 (17)	C11—H11A	0.9300
N3—C2	1.4490 (16)	C12—C13	1.3819 (19)
N4—C4	1.4546 (18)	C12—H12A	0.9300
C1—C6	1.4110 (18)	C13—H13A	0.9300
C1—C2	1.4193 (15)	C14—H14A	0.9600
C2—C3	1.3869 (18)	C14—H14B	0.9600
C3—C4	1.3678 (18)	C14—H14C	0.9600
C3—H3A	0.9300	C15—H15A	0.9600
C4—C5	1.3976 (18)	C15—H15B	0.9600
C5—C6	1.3597 (19)	C15—H15C	0.9600
C5—H5A	0.9300		
			
C9—O5—C15	118.48 (11)	C8—C7—C14	121.33 (12)
C1—N1—N2	118.56 (11)	C13—C8—C9	118.18 (12)
C1—N1—H1N1	117.2 (11)	C13—C8—C7	119.15 (11)
N2—N1—H1N1	123.6 (11)	C9—C8—C7	122.65 (12)
C7—N2—N1	117.18 (11)	O5—C9—C10	123.75 (12)
O2—N3—O1	121.81 (11)	O5—C9—C8	115.96 (11)
O2—N3—C2	118.84 (11)	C10—C9—C8	120.24 (12)
O1—N3—C2	119.35 (10)	C11—C10—C9	120.03 (13)
O3—N4—O4	122.78 (13)	C11—C10—H10A	120.0
O3—N4—C4	118.94 (13)	C9—C10—H10A	120.0
O4—N4—C4	118.27 (12)	C12—C11—C10	120.27 (13)
N1—C1—C6	119.96 (11)	C12—C11—H11A	119.9
N1—C1—C2	123.41 (11)	C10—C11—H11A	119.9
C6—C1—C2	116.63 (11)	C11—C12—C13	119.94 (13)
C3—C2—C1	121.74 (11)	C11—C12—H12A	120.0
C3—C2—N3	116.67 (10)	C13—C12—H12A	120.0
C1—C2—N3	121.58 (11)	C12—C13—C8	121.33 (13)
C4—C3—C2	118.91 (11)	C12—C13—H13A	119.3
C4—C3—H3A	120.5	C8—C13—H13A	119.3
C2—C3—H3A	120.5	C7—C14—H14A	109.5
C3—C4—C5	121.27 (12)	C7—C14—H14B	109.5
C3—C4—N4	119.79 (11)	H14A—C14—H14B	109.5
C5—C4—N4	118.94 (12)	C7—C14—H14C	109.5
C6—C5—C4	119.77 (12)	H14A—C14—H14C	109.5
C6—C5—H5A	120.1	H14B—C14—H14C	109.5
C4—C5—H5A	120.1	O5—C15—H15A	109.5
C5—C6—C1	121.68 (11)	O5—C15—H15B	109.5
C5—C6—H6A	119.2	H15A—C15—H15B	109.5
C1—C6—H6A	119.2	O5—C15—H15C	109.5
N2—C7—C8	113.69 (11)	H15A—C15—H15C	109.5
N2—C7—C14	124.87 (12)	H15B—C15—H15C	109.5
			
C1—N1—N2—C7	−175.05 (13)	N1—C1—C6—C5	179.97 (13)
N2—N1—C1—C6	−2.06 (19)	C2—C1—C6—C5	0.3 (2)
N2—N1—C1—C2	177.53 (12)	N1—N2—C7—C8	176.97 (11)
N1—C1—C2—C3	−179.48 (13)	N1—N2—C7—C14	0.9 (2)
C6—C1—C2—C3	0.13 (19)	N2—C7—C8—C13	−39.91 (17)
N1—C1—C2—N3	−0.5 (2)	C14—C7—C8—C13	136.32 (15)
C6—C1—C2—N3	179.12 (12)	N2—C7—C8—C9	138.53 (14)
O2—N3—C2—C3	2.10 (19)	C14—C7—C8—C9	−45.24 (19)
O1—N3—C2—C3	−178.19 (13)	C15—O5—C9—C10	−6.2 (2)
O2—N3—C2—C1	−176.94 (13)	C15—O5—C9—C8	176.37 (14)
O1—N3—C2—C1	2.8 (2)	C13—C8—C9—O5	177.73 (12)
C1—C2—C3—C4	−0.4 (2)	C7—C8—C9—O5	−0.73 (19)
N3—C2—C3—C4	−179.47 (12)	C13—C8—C9—C10	0.2 (2)
C2—C3—C4—C5	0.3 (2)	C7—C8—C9—C10	−178.24 (12)
C2—C3—C4—N4	−179.60 (13)	O5—C9—C10—C11	−177.64 (13)
O3—N4—C4—C3	−1.0 (2)	C8—C9—C10—C11	−0.3 (2)
O4—N4—C4—C3	179.99 (15)	C9—C10—C11—C12	0.2 (2)
O3—N4—C4—C5	179.15 (16)	C10—C11—C12—C13	0.1 (2)
O4—N4—C4—C5	0.1 (2)	C11—C12—C13—C8	−0.2 (2)
C3—C4—C5—C6	0.2 (2)	C9—C8—C13—C12	0.0 (2)
N4—C4—C5—C6	−179.93 (14)	C7—C8—C13—C12	178.55 (13)
C4—C5—C6—C1	−0.5 (2)		

### Hydrogen-bond geometry (Å, °)

**Table:**

*D*—H···*A*	*D*—H	H···*A*	*D*···*A*	*D*—H···*A*
N1—H1N1···O1	0.87 (2)	1.952 (18)	2.6086 (17)	131.1 (15)
C6—H6A···O3^i^	0.93	2.48	3.218 (2)	136

Symmetry codes:  (i) *x*, −*y*, *z*−1/2.
